# Fletcherism, and Some of Its Effects on Nutrition

**Published:** 1910-09-15

**Authors:** John B. West

**Affiliations:** Groton, N. Y.


### FLETCHERISM, AND SOME OF ITS EFFECTS ON NUTRITION.

BY JOHN B. WEST, D.D.S., GROTON, N. Y.

Some years ago Mr. Horace Fletcher, an American, ready to retire from active business life, made application for life insurance and was refused. His symptoms were obesity, shortness of breath, dyspepsia, loss of elasticity and keenness; in short, all those troubles that we are accustomed to associate with advancing years. He consulted medical men both in America and Europe, but without receiving any help. One summer, being in Chicago on some business which required a great deal of tedious waiting, he used to get through his meals as slowly as possible to help pass away the day. He observed several curious effects from this—hunger was less frequent, he ate less, his weight decreased, and in the course of time he entirely recovered his health. The Physiological discovery made by Mr. Fletcher has come to be called, not inappropriately, "Fletcherism."

Mr. Fletcher's method of taking nutrition may be expressed in two rules: (1) Mastication, and (2) Following the instinct regarding the special senses of taste and appetite.

(1) *Mastication.* By mastication Fletcher means thorough mastication of food to the point of involuntary swallowing, the attention not being fixed on the mechanical act of chewing, but on the tasting and enjoying of food, liquid food being sipped and tasted, not drunk down like water. There should be no forcing or holding of food toward the anterior part of the mouth, or counting the chews or allowing the tongue muscles to become fatigued by any unnatural effort or position, or in any way making eating a bore. On the contrary, one should be in a happy frame of mind and thoroughly enjoy his food, for Pawlow has shown that without such attention and enjoyment of food the secretion of gastric juice is lessened. To quote from Prcfesser Irving Fisher: "The point of involuntary swallowing is thus a variable point, gradually coming later and later as the practice of thorough masticatication proceeds until the result is reached that the food remains in the mouth without effort and becomes practically tasteless. Thus the food, so to speak, swallows itself, and the person eats without thought either of swallowing or not swallowing it; swallowing is put in the same category of physiological functions as breathing—which ordinarily is involuntary."

Since Dr. Ernest Van Somern of Venice, Italy, read a paper before the British Medical Association in 1901 on the principles of nutrition as advanced by Horace Fletcher, some of the greatest physiological experts in the world have been investigating human nutrition. Two of these experts Dr. Hubert Higgins, formerly demonstrator of anatomy at the university of Cambridge, Eng., and Professor Paul Heger, professor of physiology in the university of Brussels, undertook anew the study of the first three inches of the alimentary canal of man, giving scientific support to Mr. Fletcher's ideas on mastication and insalivation. Dr. Higgins and Professor Heger found that Gegenbaur, a famous German anatomist, had made a statement in his writings that had passed unnoticed by both anatomists and physiologists until Mr. Fletcher stirred up interest in this subject. On page 90 of Gegenbaur's Anatomy ("Anatomie der Wirbelthiere") the following passage is found: "the bifurcation of the alimentary canal below the soft palate does not depend only on its relation with the epiglotis, but also on the condition of the food. The exclusive use of this means of swallowing is only possible with finely divided food—I have called this way of taking food POLTOPHAGY (*poltos*,masticated, finely divided), and the other, PSOMOPHAGY (*psomos*, biting, tearing)."

By a series of experiments Dr. Higgins and Professor Heger found that the muscles of the soft palate could produce both a negative and positive pressure, and that the soft palate is capable of making the mouth cavity airtight. When a mouthful is swallowed in a gulp, the soft palate has a sustained backward movement, an instance of psomophagic swallowing, and when the food is finely masticated, there is a forward movement of the soft palate, an instance of poltophagic swallowing. Thus man has both a psomophagic and a poltophagic faculty. Dr Higgins Remarks, "In the case of the anthropoid apes and man there has been a shortening of the upper jaw from before backward, on account of the assumption of the erect posture, which has resulted in the soft palate becoming more vertical, and thus enabling psomophagic deglutition to be practiced." To recapitulate, Mr. Fletcher has drawn attention to a human faculty that was not recognized. If this process is used to its full extent, a certain series of phenomena result, some of which we will consider in this paper.

(2) *Following instinct.* Never eat when not hungery even if several meals are skipped befor you have a normal appetite. When taking a meal one should not be guided by the quantity of food offered, or by past habits, or any theories as to the amount of food needed. The natural appetite alone is consulted, and one selects from the food available only those kinds and amounts that are actually craved by the appetite. Thus the body selects through normal appetite the food that it requires.

Appetite had been somewhat over looked by physiologists on account of the observations of Dr. Beaumont in the celebrated case of St. Martin, who had an aperture made in his stomach as the result of a gunshot wound. These oberservations were thought to show that the stimulation for the secretion of gastric juice was mere contact of the food with the wall of the stomach. The contact stimulous for the flow of gastric juice has been disproved by the researches of professor Pawlow, the eminent Russian physiologist. The experiments of Pawlow prove that Fletcher is correct in the value he places on appetite and taste in feeding. Professor Pawlow says in summing up his conclusions: "It is only by the establishment of this passionate desire for eating that unerring and untiring nature has linked the seeking and finding of food with the commencement of the work of digestion. That this factor, which we now have so carefully analyzed, stands in closest relation with a phenomenon of daily life, namely appetite, may easily be predicated. That agency which is so important to life and so full of mystery to science becomes at length incorporated into flesh and blood, transformed from a subjective sensation into a concrete factor of the physiological laboratory. We are justified in saying that appetite is the first and mightiest exciter of the secretory nerves of the stomach.

Mr. Fletcher lays strong emphasis on taste as the guide and guard of nutrition. He says: "Taste, in its normal condition, when allowed to direct or advise, serves several important functions, not the least of which is at first assistant to appetite. Appetite craves the kind of nourishment the body needs, invites to eating, gives enjoyment during the whole time needed for the fluids of the mouth and stomach to do their part of the digestive process. Taste ceases when the food is ready for the stomach and thereafter fails to recognize the indigestible sediment which remains in the mouth after nutriment has been extracted; and in these discriminations, if consulted and obeyed, taste and appetite prevent indigestible matter from entering the system to burden and to clog the lower intestines, form deposits in bone, cartilage, and kidneys inflame the tissues and otherwise create conditions favorable to the propagation of the microbes of disease."

### SOME EFFECTS OF FLETCHERISM ON NUTRITION.

The practice of Fletcherism produces marked changes in nutrition. Less food is taken, the appetite becomes more discriminating, and meales are reduced to one or two .per day.

From January to June 1906, at Yale University, Professor Irving Fisher conducted with nine healthy students an experiment in thorough mastication and instinctive eating to determine the effect of diet on endurance. "At the outset, the men consumed daily an average of 2830 calories. A Calory is the amount of heat required to raise the temperature of one kilogram of water 1° C. Of these 2830 calories, 210 were in the form of flesh foods, such as meats, poultry, fish, and shell-fish, 2.6 calories of proteid being ingested for each pound of body weight. At the close of the experiment, the per capita calories had fallen to 2220, of which only 30 were in flesh foods, and the proteid had fallen to 1.4 calories per pound of body weight. In other words, the total calories of the daily ration had dropped off about 25 per cent., the proteid about 40 per cent., and the flesh foods over 80 per cent.,or to about one sixth of their original amount.

When Fletcher first advanced his ideas he found that physiology had a considerable bogy to confront him with called the Voit standard of nitrogenous nutrition. He was informed that before he could obtain acceptance of his claims he must prove his standard of nutrition to be nearer the normal than the famous Voit standard.

Mr. Fletcher was fortunate enough to find a practical sympathizer in Professor Russell H. Chittenden of Yale University, who is one of the most eminent of physiological chemists. Professor Chittenden found that during an experiment of seven days, Mr. Fletcher consumed an average of only 45 grams of proteid per day, and that fats and carbohydrates were taken in quantities only sufficient to bring the total fuel value up to a little more than 1600 large calories. These figures speak for themselves when we recall that the Voit standard demand at least 118 grams of proteid and a total fuel value of 2000 large calories daily. It may be said in explanation of this experiment that the body weight remained practically constant during the seven days'period and that the intake and output of nitrogen were not far apart; in other words, there was a close approach to what physiologists call a nitrogenous equilibrium. The question might be asked: Could a man on such a diet, even though it suffice to keep up body weight and physiological equilibrium, do work to any extent? In reply to such a possible question, Mr. Fletcher was given the same kind of exercises that are given the 'varsity crew at Yale. Dr. W. G. Anderson, director of the Yale gymnasium, in speaking of this experiment, gives his conclusions in the following words: "Mr. Fletcher performed this work with greater ease and with fewer noticeable bad results than any man of his age and condition I have ever worked with.

We have gone into details concerning this experiment, since the result is the same as that of the more elaborate test made by Professor Chittenden on students, athletes, and soldiers of the United States army. By these extensive and laborious researches of Professor Chittenden it is proved that men can play better, study better, work better, and have better health, not on the Voit standard, but on one-half or one-third the amount of nitrogen prescribed therein as being essential.

In Conclusion, those who practice the rational and natural method of taking their nutrition as advocated by Mr. Fletcher, observe nearly the same phenomena as professor Fisher observed at Yale as the result of the experiment to which we have already referred. There is a slight reduction of the total food consumed, a large reduction of the proteid element, especially of flesh foods, a lessened excretion of nitrogen, a reduction in the odor putrefaction, fermentation, and quantity of the feces, a slight loss of strength, an enormous increase of physical endurance, a slight increase in mental quickness. These phenomena vary somewhat in different individuals, the variations corresponding, in general, to the thoroughness with which one carries out the ideas of this method of nutrition.—*Cosmos.*

